# Responsive Hydrogel-Based Drug Delivery Platform for Osteoarthritis Treatment

**DOI:** 10.3390/gels10110696

**Published:** 2024-10-26

**Authors:** Bin Yin, Jianda Xu, Jingqi Lu, Changjin Ou, Kai Zhang, Fan Gao, Yizhou Zhang

**Affiliations:** 1 Institute of Advanced Materials and Flexible Electronics (IAMFE), School of Chemistry and Materials Science, Nanjing University of Information Science and Technology, Nanjing 210044, China; bohemian0224@163.com (B.Y.); ljq_199802@163.com (J.L.); kaizhang@nuist.edu.cn (K.Z.); 003335@nuist.edu.cn (F.G.); 2 Nanjing University of Chinese Medicine, 25 North Heping Road, Changzhou 213003, China; milidoc3@163.com

**Keywords:** injectable hydrogel, osteoarthritis, drug delivery, stimuli responsive

## Abstract

Osteoarthritis (OA) is the most prevalent chronic joint disorder and is a major cause of disability among the elderly population. The degeneration and damage of articular cartilage associated with OA can result in a diminished range of motion in joints, subsequently impacting fundamental activities such as ambulation, standing, and grasping objects. In severe cases, it may culminate in disability. Traditional pharmacological treatments are often accompanied by various side effects, while invasive surgical procedures increase the risk of infection and thrombosis. Consequently, identifying alternative new methods for OA treatment remains a formidable challenge. With advancements in responsive hydrogel drug delivery platforms, an increasing number of strategies have emerged to enhance OA treatment protocols. Injectable response hydrogel drug delivery platforms show many advantages in treating OA, including improved biocompatibility, prolonged drug release duration, elevated drug loading capacity and enhanced sensitivity. This article reviews the recent progress of injectable responsive hydrogel drug delivery platform for OA treatment over the past few years. These innovative methodologies present new strategies and directions for future OA treatment while summarizing a series of challenges faced during the clinical transformation of injectable response hydrogel drug delivery platforms. Overall, injectable responsive hydrogel drug delivery platforms show great potential in treating OA, especially regarding improving drug retention time and stimulus-responsive release at the lesion sites. These innovative methods provide new hope for future OA treatment and point the way for clinical applications.

## 1. Introduction

Osteoarthritis (OA), one of the most prevalent chronic diseases, is a degenerative joint disorder with etiological factors including joint injury, obesity, aging, and genetics. It is estimated that approximately 500 million individuals worldwide are affected by OA, accounting for 7% of the overall disease prevalence [1,2]. Notably, this proportion escalates to 30% among the elderly population and postmenopausal women [1,2]. Major clinical symptoms include chronic pain, joint instability, stiffness, and radiographic joint space narrowing [3]. The pathogenesis of OA involves multiple factors, including mechanical effects, the impact of aging on the composition and structure of the cartilage matrix, as well as genetic factors [4]. In the early stages of OA progression, chondrocytes promote cell proliferation and synthesize matrix proteins, proteases, growth factors, cytokines, and other inflammatory mediators. Consequently, the research interests predominantly focus on chondrocytes as key mediators of OA pathogenesis [5]. Specifically speaking, excessive recruitment of inflammatory cells to the synovial area reduces the synthesis of type II collagen (COL2) and proteoglycans. Simultaneously, overexpression of matrix-degrading enzymes, including matrix metalloproteases (MMPs) [6] and A disintegrin and metalloproteinases with thrombospondin motifs (ADAMTS) [7], leads to the degradation of the extracellular matrix (ECM) and subsequent cartilage destruction [8,9].

Although the treatment strategy for OA aims to alleviate pain, reduce disease activity, and prevent inflammation and destructive processes, a complete cure for the disease remains elusive [10]. For example, non-steroidal anti-inflammatory drugs (NSAIDs) and opioid drugs are frequently used for OA management, but they can induce a series of side effects that limit their clinical application [11]. Invasive surgical procedures, including arthroplasty, increase the risk of infection and thrombosis, particularly for elderly patients [12]. Platelet-rich plasma (PRP) [13] and mesenchymal stem cells (MSCs) [14] have been administered via injection into the joint cavity for the treatment of OA, only a limited number of these cells survive or remain in situ. Intra-articular injection therapy is an attractive strategy for the local treatment of joint diseases [15], which gained popularity in the latter half of the 20th century [16,17]. While intra-articular injections into synovial fluid offers advantages such as high intra-articular drug concentration and minimal side effects compared to traditional methods, several issues have to be surmounted before clinical application. Various uncontrollable factors, such as poor drug permeability in cartilage, rapid clearance of components through synovial capillaries and lymphatic vessels, and weakened synergy of active ingredients in the pathological microenvironment, limit the effectiveness of intra-articular administration [15]. Therefore, there is an urgent need to explore innovative drug delivery systems to improve the therapeutic outcomes in OA.

Hydrogel is a three-dimensional (3D) cross-linked polymer network capable of absorbing a large amount of water or biological fluid without dissolving. This characteristic renders it a promising platform for various biomedical applications, including drug delivery, cell culture, and tissue engineering [18]. In the context of OA, hydrogels provide support to damaged cartilage, alleviate pressure on affected areas, and slow down the progression of the disease [19]. Notably, injectable hydrogels exist in liquid form for injection and then transform into a solid gel after being injected into lesion sites [20].

**Scheme 1:**
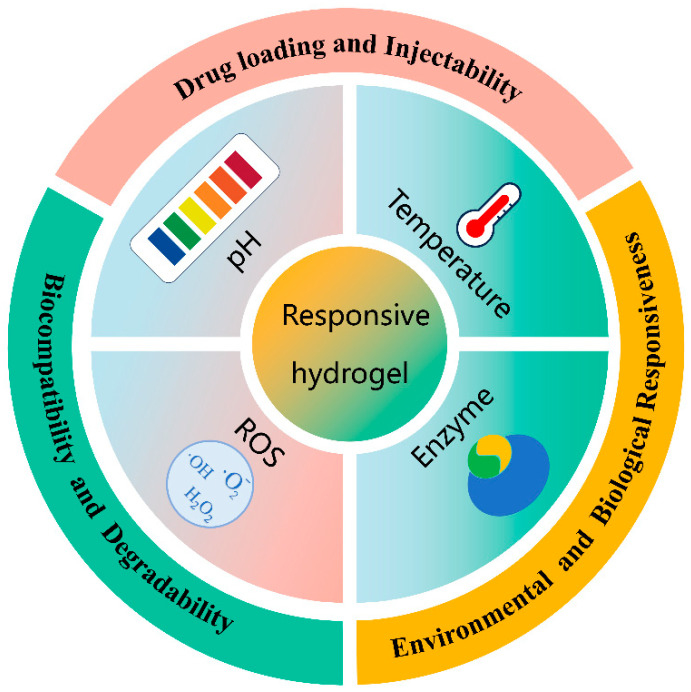
Responsive factors and their properties of hydrogels for OA treatment.

Injectable responsive hydrogel drug delivery platforms demonstrate significant potential in enhancing the therapeutic outcomes of OA treatment. Their capacity to exhibit ideal physical and chemical properties, combined with their excellent biocompatibility and biodegradability, significantly broadens their application in drug therapy [21]. Moreover, stimulation-responsive hydrogel platforms have emerged [22], which can controllably induce drug release based on various stimuli within the organism, such as enzymes, reactive oxygen species (ROS), pH, and temperature (Scheme 1).

Hydrogels have emerged as highly promising candidates for advancing the treatment of OA due to their inherent biocompatibility, customizable attributes, and adaptability in design. However, these materials face several substantial limitations and technical challenges in their clinical translation to widespread therapeutic use, necessitating careful consideration and innovative solutions. One of the primary challenges in utilizing polymeric biomaterials for OA treatment is achieving mechanical properties that can withstand the physiological loading conditions within the joint. An ideal hydrogel should provide robust support and stability while maintaining structural integrity over extended periods. This challenge remains critical, demanding the development of materials capable of enduring the complex mechanical stresses encountered in the joint [23].

Combining 3D printing with hydrogel fabrication may hold the key to addressing this issue. If a slow-release system for artificial materials can be constructed to facilitate the gradual release of various drugs, such as antibiotics, anti-inflammatory analgesics, and cartilage regeneration agents, it would not only create an optimal environment for cartilage repair but also inhibit the progression of OA. This approach is likely to lead to significant improvements in postoperative functional recovery and patient satisfaction, ultimately enhancing patient outcomes [24].

In addition, addressing the issue of sudden drug release from hydrogels remains a formidable challenge for current injectable hydrogel systems [25]. The microenvironment of OA affected joints is complex, involving inflammatory factors, oxidative stress, enzymatic reactions, and other factors that may accelerate the degradation of hydrogels or affect their drug release performance. However, these various signals can also be leveraged to improve the degradation, sustained release, and controlled release of hydrogels. Therefore, hydrogels must maintain stability in this complex environment to ensure the predictability and consistency of drug release profiles.

This article reviews the use of injectable responsive hydrogels in the treatment of OA in recent years, highlighting hydrogels that respond to various stimuli and their corresponding therapeutic strategies (Table 1). The goal is to provide a reference for the development of novel injectable responsive hydrogels, offering insights into their potential applications in OA therapy.

## 2. The Microenvironment-Responsive Hydrogels for OA

The microenvironment of OA is characterized by the abnormal expression of degradative enzymes, redox imbalances, fluctuations in pH levels and variation in temperature. The etiology of OA is multifactorial, driven by mechanical factors such as overuse, joint instability, or injury. These factors contribute to disease progression by triggering cartilage damage and inflammatory responses. The design of responsive hydrogel carriers targeting specific factors has the potential to improve therapeutic efficacy while reducing side effects on normal tissues. This section will explore the development of hydrogel-based drug delivery systems that respond to endogenous stimuli, highlighting their practical applications and potential for treating OA.

### 2.1. pH-Responsive Hydrogel

Extensive research has demonstrated that the pH of biological tissues can deviate from the normal levels, especially in disease or injury situations. Inflammatory sites exhibit altered pH compared to normal tissue. When inflammation begins, the pH value decreases. After the inflammation stabilizes, the pH value remains acidity [38]. For instance, the pH of the joint lubricant in arthritis ranges from 6.6 to 7.2, whereas normal synovial fluid ranges from 7.4 to 7.8 [39]. 

This acidic characteristic (pH < 7.2) is believed to be associated with increased energy and oxygen consumption by infiltrating inflammatory cells, causing a metabolic shift in inflammatory tissues from aerobic to anaerobic processes, which results in increased glycolysis and lactate accumulation [40]. Consequently, this pH-responsive drug delivery platform can be exploited based on the pathological acidity differences in the microenvironment at the OA joint site to enhance drug delivery effectiveness and feasibility in OA treatment [41]. To improve the OA treatment outcomes, researchers are developing injectable hydrogels that incorporate pH-sensitive materials along with anti-inflammatory drugs or other therapeutic agents. 

**Figure 1:**
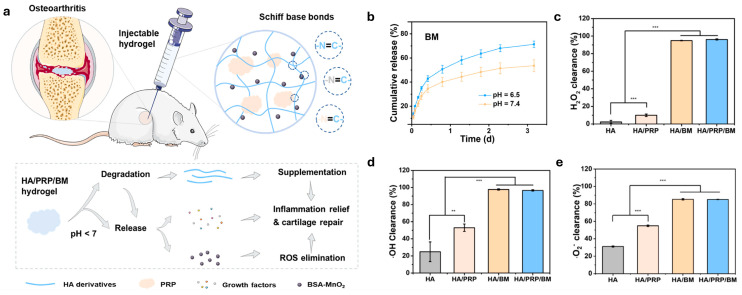
(**a**) Schematic illustration showing the injectable hydrogel of HA/PRP/BM fabricated via Schiff base reaction, and its synergetic treatment of OA owing to viscosupplementation, ROS elimination, inflammation relief, and cartilage repair promotion. (**b**) The cumulative release profile of BM from HA/BM hydrogel in PBS with different pH values. Depletion test of hydrogels for (**c**) hydrogen peroxide (H_2_O_2_), (**d**) hydroxyl radical (·OH), and (**e**) superoxide anion radical (O_2_) (** *p* < 0.01 and *** *p* < 0.001) [26]. Copyright 2022 Elsevier.

Currently, the primary reaction mechanisms of pH-sensitive materials include acid-sensitive bond cleavage and protonation of chemical groups [42]. The former involves introducing acid-sensitive bonds into polymer structures, serving as connectors between drugs and carriers. Consequently, the release of drug molecules is achieved by breaking these acid-sensitive bonds or reversing the charge of polymers (such as hydrazine, vinyl ester, and imine) in response to pH changes in the surrounding environment, thereby promoting drug entry into target cells [43]. For example, Zhou et al. developed a hydrogel network combining hyaluronic acid (HA) and PRP, coated with bovine serum albumin (BSA) and manganese dioxide (BM) nanoparticles. The active aldehyde group on oxidized hyaluronic acid (HA-ALH) reacts with the amino group on adipic hydrazide-grafted hyaluronic acid (HA-ADH) to form a Schiff base, rendering the hydrogel pH-responsive. The growth factors in PRP simultaneously provide anti-inflammatory effects and stimulate functions such as cartilage matrix synthesis, bone remodeling, wound healing, and cytokine metabolism (Figure 1a). In vitro experiments showed that the hydrogels remained stable under normal physiological condition, while the inflammatory environment was capable of boosting the release rate (Figure 1b). Such an encapsulation system could meet the requirements of on-demand delivery of BM NPs and growth factors in the OA microenvironment. Additionally, BSA-BM NPs not only have antioxidant properties, but also exhibit a rapid scavenging ability against various types of ROS, including superoxide anions (·O_2_) and hydroxyl radicals (·OH) (Figure 1c–e) [26]. 

In contrast to the cleavage of pH-sensitive chemical bonds, the protonation mechanism involves materials that can be protonated in acidic microenvironment of OA. Protonation of nanoscale materials often leads to changes in properties such as stability, surface charge, and morphology. For example, cationic polymers can expand upon protonation in acidic conditions, facilitating the release of drugs sequestered in cartilage tissue. pH-sensitive polymers, peptides, and inorganic materials (including metals and non-metals) are used to create pH-sensitive drug delivery systems based on protonation mechanisms [44].

**Figure 2:**
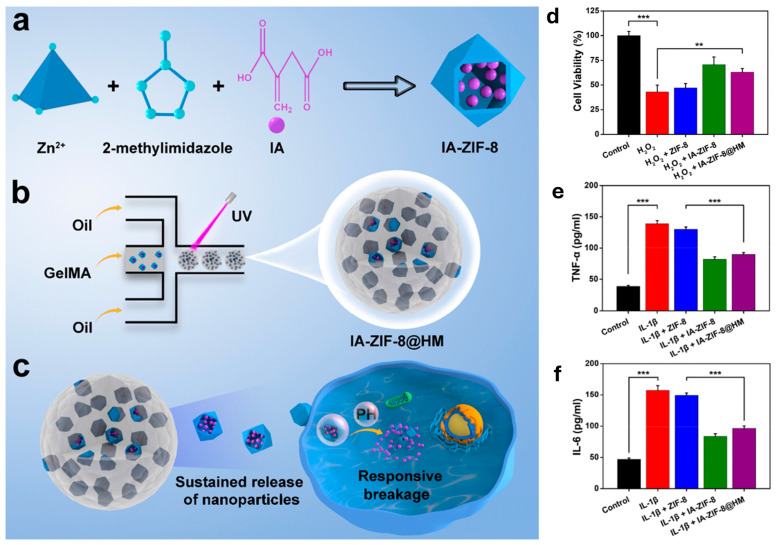
(**a**) The synthesis of the itaconate (IA)-encapsulated zeolitic imidazolate framework-8 (IA-ZIF-8) nanoparticles. (**b**) The fabrication of IA-ZIF-8-loaded hydrogel microspheres (IA-ZIF-8@HMs) by one-step microfluidic technology under ultraviolet (UV) light. (**c**) The design of IA-ZIF-8@HMs for treating OA. (**d**) The results of cell viability about chondrocyte co-cultured with H_2_O_2_, H_2_O_2_ + ZIF-8, H_2_O_2_ + IA-ZIF-8, and H_2_O_2_ + IA-ZIF-8@HM. Enzyme-linked immunosorbent assay (ELISA) analyses of TNF-α (**e**) and IL-6 (**f**) concentration in cell supernatants (** *p* < 0.01, *** *p* < 0.001) [27]. Copyright 2023 Multidisciplinary Digital Publishing Institute.

Yu et al. developed a ZIF-8 nanoparticle hydrogel microsphere (IA-ZIF-8@HMs) coated with itaconic acid (IA), which exhibited both pH responsiveness and protonated acid responsiveness. IA can regulate joint inflammation and intracellular oxidative stress. The injectable GelMA hydrogel microspheres (HMS) were prepared using one-step microfluidic technology, resulting in highly dispersed and uniformly size. Due to their micron size, these microspheres can remain suspended in synovial fluid for an extended period (Figure 2a–c). In vitro experiments indicated that IA-ZIF-8@HMs could effectively inhibit H_2_O_2_-induced oxidative stress, and the cell-impermeable of IA might be trafficked into chondrocytes through the composites in this study (Figure 2d). After being treated with IA-ZIF-8 nanoparticles and IA-ZIF-8@HMs, the levels of inflammation factors exhibited significant drops (Figure 2e,f). These data suggested that IA-ZIF-8@HMs could effectively suppress inflammation and are promising for OA therapy in vivo [27].

### 2.2. ROS-Responsive Hydrogel

ROS, as byproducts of cellular metabolism, are highly active molecules. The effects of ROS on cells vary depending on their levels and concentrations. At low level, ROS can act as signaling molecules to maintain physiological conditions, activate downstream membrane receptors, and sustain normal cell function [45,46]. However, elevated ROS levels result from an imbalance between ROS production and the antioxidant system, leading to oxidative stress. Excessive accumulation of ROS can cause cellular dysfunction and inflammation [47,48,49]. Differences in ROS levels exist between normal and pathological tissues [50], particularly in conditions such as tumors and inflammation.

ROS can accumulate abnormally at pathological sites, making them ideal markers for actively targeting and inducing controlled drug release [51]. ROS-responsive materials undergo changes in high ROS environments, such as alterations in hydrophilicity, hydrophobicity, and fracture. These responsive polymer materials exhibit high modifiability and selectivity, enabling them to release drugs at different ROS levels. This allows for precise local treatment, reduces drug damage to normal tissues, and enhances drug accumulation in joint areas, thereby improving treatment effectiveness. ROS-responsive materials mainly include sulfur-based, selenium-based, tellurium-based responsive polymers, as well as phenylboronic acid-based polymers [52].

**Figure 3:**
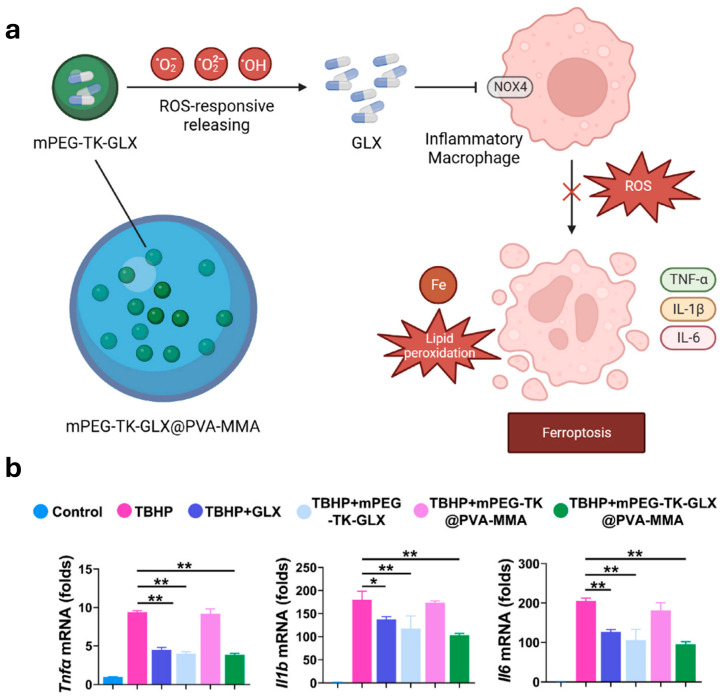
(**a**) Schematic representation of the potential application of mPEG-TK-GLX@PVA-MMA to mitigate macrophage inflammation and ferroptosis. (**b**) Expression of pro-inflammatory genes evaluated by RT-qPCR (* *p* < 0.05 and ** *p* < 0.01) [28]. Copyright 2024 Elsevier.

Zhen et al. prepared polyethylene glycol ketone mercaptan (mPEG-TK) containing disulfide bonds and a new selective NOX4 inhibitor, GLX351322 (GLX), to form nanoparticles (mPEG-TK-GLX) by self-assembly. These nanoparticles were then loaded onto polyvinyl acetate-methyl methacrylate (PVA-MMA) injectable HMS (Figure 3a). Disulfide bonds are typical ROS-responsive groups; when ROS concentrations are high, these bonds are oxidized and broken, releasing the encapsulated GLX. NADPH oxidase 4 (NOX4) is one of the sources of ROS, and GLX is an inhibitor of NOX4, effectively reducing ROS generation. The RT-qPCR results (Figure 3b) illustrated that the mRNA levels of pro-inflammatory genes, including Tnf, Il1b, and Il6, were upregulated with TBHP (Tert-Butyl Hydroperoxide) stimulation, while markedly reduced after treatment with GLX, mPEG-TK-GLX or mPEG-TK-GLX@PVA-MMA. Notably, the treatment of mPEG-TK-GLX or mPEG-TK-GLX@PVAMMA exhibited a better inhibitory effect on pro-inflammatory genes than the treatment GLX. The mPEG-TK-GLX@PVA-MMA system exerted the high ROS levels in the inflammatory microenvironment to activate the nanoparticles, accelerated the release of the target drug GLX, improved the cellular inflammatory microenvironment, reduced ROS generation, and alleviated oxidative stress and iron deficiency [28].

**Figure 4:**
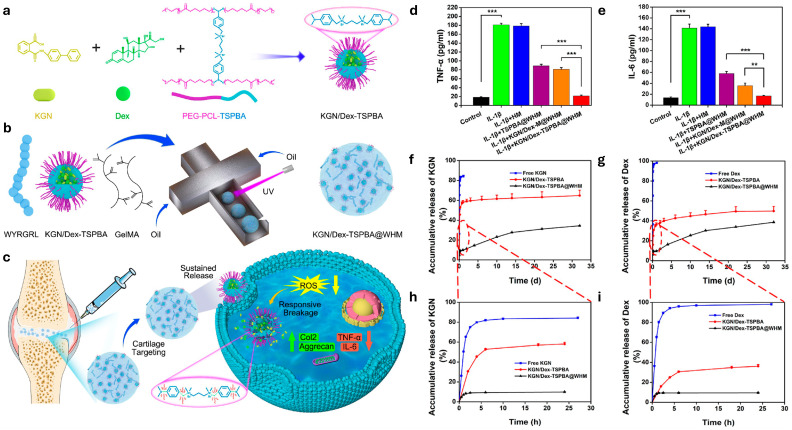
(**a**) The fabrication of the KGN/Dex-TSPBA nanoparticles. (**b**) The fabrication of KGN/Dex-TSPBA@WHMs. (**c**) The mechanism of KGN/ Dex-TSPBA@WHMs in the treatment of OA. The levels of (**d**) TNF-α and (**e**) IL-6 secreted from ATDC5 cells after being treated with PBS, IL-1β, IL-1β+HM, IL-1β+TSPBA@WHMs, IL-1β+KGN/Dex-M@WHMs, and IL1β+KGN/Dex-TSPBA@WHMs. (**f**) Release behavior of KGN from free KGN, KGN/Dex-TSPBA, and KGN/Dex-TSPBA@ WHMs in PBS for 32 days. (**g**) Release behavior of Dex from free Dex, KGN/Dex-TSPBA, and KGN/Dex-TSPBA@WHMs in PBS for 32 days. (**h**) Release behavior of KGN from free KGN, KGN/Dex-TSPBA, and KGN/Dex-TSPBA@WHMs in PBS in the first 24 h. (**i**) Release behavior of Dex from free Dex, KGN/Dex-TSPBA, and KGN/Dex-TSPBA@WHMs in PBS in the first 24 h (** *p* < 0.01, *** *p* < 0.001) [29]. Copyright 2020 BioMed Central.

Other ROS-responsive hydrogels were also fabricated to enhance therapeutic efficacy of OA. Yu et al. developed injectable HMS KGN (kartogenin)/Dex (dexamethasone)-TSPBA@ WHMs. The cartilage-targeting peptide WYRGRL and ROS-responsive nanoparticles KGN/Dex-TSPBA were encapsulated and anchored in a GelMA hydrogel matrix using microfluidic technology. The phenylboronic acid bond (PBA) in the HMS responds to ROS for drug release. KGN can promote the differentiation of stem cells into cartilage, while Dex alleviates inflammation and reduces cartilage degradation. The WYRGRL targeting peptide enhances the cartilage-targeting ability of nanoparticles, allowing therapeutic drugs to accurately treat cartilage (Figure 4a–c). Experimental results indicated that KGN/Dex-TSPBA@WHMs not only specifically targeted type COL2 with the help of WYRGRL but also exhibited excellent ROS clearance properties in vitro and ROS-responsive release of both drugs to alleviate inflammation (Figure 4d,e). Additionally, KGN/Dex-TSPBA@WHMs showed a good sustained-release ability (Figure 4f–i), prolonging drug retention due to KGN/Dex and promoting the repair of damaged cartilage in vivo. KGN/Dex-TSPBA@WHMs provided a new strategy for exploring innovative treatments for OA [29].

In addition, Liu et al. designed a ROS-responsive hydrogel composed of APBA (3-aminophenylbutyric acid)-SF (modified silk fibroin protein)-PVA (polyvinyl alcohol), loaded with interferon IFN-γ-loaded microvesicles (MVS) to stimulate MSCs. APBA is grafted onto PVA to form borate ester bonds responsive to ROS, enabling on-demand drug release based on ROS concentrations at bone and joint sites. Studies indicate that stem cell therapy operates via paracrine pathways rather than requiring direct differentiation and replacement [53].

**Figure 5:**
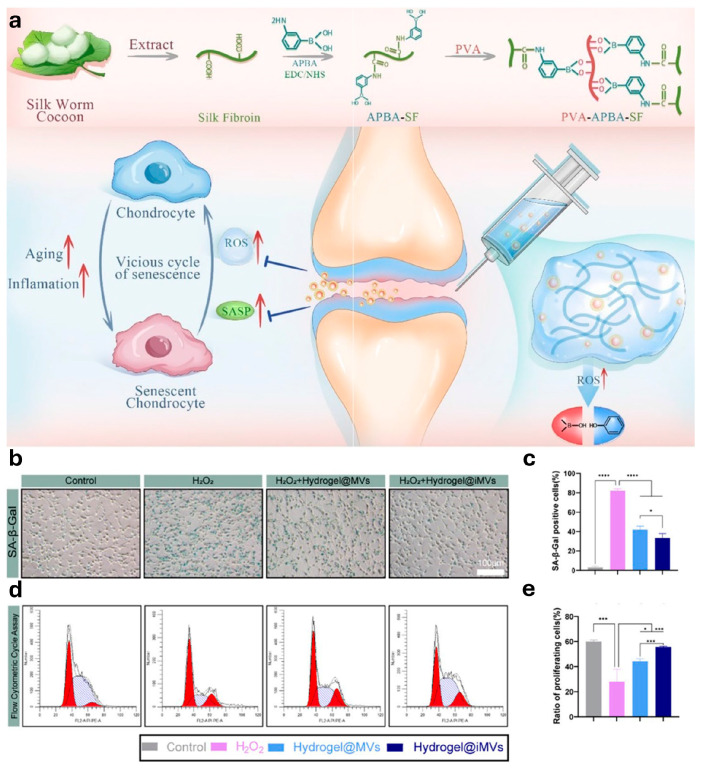
(**a**) The principle and production of anti-ageing Hydrogels@MVs/iMVs43. (**b**,**c**) SA-β-Gal staining and percentage of senescent cells after ageing induction and intervention with different factors. (**d**,**e**) Cell cycle analysis and percentage of proliferating cells after the same treatment using flow cytometry (* *p* < 0.05, *** *p* < 0.001, **** *p* < 0.0001) [30]. Copyright 2023 BioMed Central.

MVS secreted by bone marrow stem cells can improve cellular metabolism by regulating mitochondria. Interferon IFN-γ, an important cytokine in aging cells, induces stem cells to produce MVS for repairing mitochondria and treating OA. In vitro experiments have confirmed that the hydrogel loaded with IFN-γ-MVS promoted the expression of mitochondrial fusion proteins, suppressed excessive division, and effectively improved chondrocyte aging and cartilage degeneration (Figure 5a). To assess the therapeutic potential of MVs/iMVs on chondrocyte senescence in vitro, ATDC5 cells were first induced to age using H_2_O_2_, followed by treatment with Hydrogel@MVs/iMVs. The analysis of β-galactosidase activity, a marker of cellular senescence, showed that MVs/iMVs significantly reduced the proportion of senescent cells from approximately 80% to around 40% (Figure 5b,c). Furthermore, cell cycle analysis under the same conditions demonstrated that cells treated with Hydrogel@MVs/iMVs exhibited a higher percentage of cells in the proliferative phase compared to the untreated group (Figure 5d,e). This new ROS-responsive IFN-γ-MVS hydrogel system offers a promising approach to alleviate OA chondrocyte aging [30].

### 2.3. Thermo-Responsive Hydrogel

The temperature of a healthy knee is typically lower than 37 °C, the lowest among the lower limbs. However, many reports indicate that the temperature of the articular cartilage in OA patients often exhibits a slight increase (approximately 3 °C), correlating with the severity of OA [54]. Generally, to deliver drugs into locally heated tissues, thermal-responsive hydrogels should remain stable in normal tissues at 37 °C but exhibit sensitivity and responsiveness to slight temperature changes, transitioning from hydrophilic to hydrophobic states [55]. However, it is hard to precisely control phase change within such small temperature range.

This temperature change creates opportunities for a thermo-responsive drug delivery platform for OA treatment. The primary mechanism of thermo-responsive hydrogels relies on existing thermally sensitive groups. When the temperature at the lesion site reaches the gelation temperature, thermosensitive hydrogel formation occurs instantly through micro mechanisms, facilitating the controlled release of loaded drugs at specific locations [56].

An ideal thermosensitive hydrogel delivery system should flow freely at ambient temperatures and transition into a non-flowing gel at physiological temperatures (32 °C to 37 °C) [57]. The primary components of thermo-responsive hydrogels are temperature-sensitive materials, including poly(N-isopropylacrylamide) (PNIPAM), agarose (AG), chitosan (CS), poloxamer, and pluronic F127. Generally, these substances must remain stable in healthy tissues (37 °C) while being responsive to temperature fluctuations [58].

**Figure 6:**
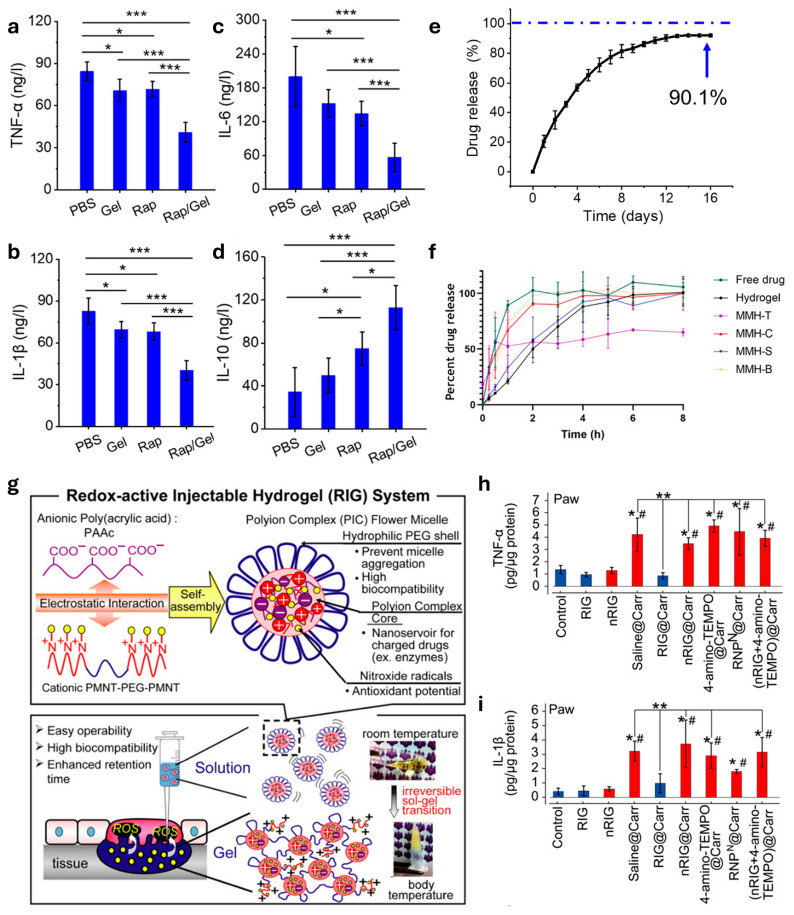
(**a**–**c**) Concentration of M1-secreted pro-inflammatory cytokines (including TNF-a, IL-1b, and IL-6) in the synovium tissues by ELISA. (**d**) Concentration of M2-secreted anti-inflammatory cytokines (IL-10) in the synovium tissues by ELISA. (**e**) Release profiles of rapamycin incorporated in the P-HA hydrogel (* *p* < 0.05, *** *p* < 0.001) [31]. Copyright 2023 Elsevier. (**f**) Hydrocortisone accumulated release-time for standard solution, base formulation, and formulations of MMH-T, MMH-C, MMH-S, and MMH-B [32]. Copyright 2023 Elsevier. (**g**) Schematic illustration of redox-active injectable hydrogel (RIG) system. Concentration of (**h**) tumor necrosis factor α (TNF-α) and (**i**) interleukin 1β (IL-1β) in the hind paw tissue of mice (* *p* < 0.05 as compared with control. # *p* < 0.05 as compared with RIG. ** *p* < 0.05 as compared with RIG@Carr.) [33]. Copyright 2013 Elsevier.

Chen et al. utilized poloxamer 407 and HA as the thermal response platform to create an injectable thermo-responsive hydrogel loaded with rapamycin. The P-HA hydrogel remained in a solution state at 4 °C before injection and transformed into a gel state at 37 °C upon injection into the joint. Subsequently, it released rapamycin continuously as needed (Figure 6e), which effectively induced a polarization in macrophage phenotype from M1 pro-inflammatory to M2 anti-inflammatory expression. This strategy significantly reduced the expression of pro-inflammatory cytokines (such as TNF-α, IL-1β, and IL-6) in synovial tissue and synovial fluid (Figure 6a–c), while increasing the expression of anti-inflammatory cytokines (such as IL-10) (Figure 6d). Therefore, intra-articular injection of rapamycin-doped P-HA hydrogel could effectively prevent cartilage destruction by promoting the formation of cartilage matrix, highlighting its potential in the treatment of OA [31].

In addition, Sheida Jahanbekam et al. designed an injectable thermo-responsive hydrogel using Pluronic F-127, HA, and gelatin for drug-loaded aqueous gel delivery. They prepared an in situ hydrogel loaded with hydrocortisone and optimized the process using Design Expert software. The optimized hydrogel was further compounded with four different surfactants to enhance control over drug release rates (Figure 6f). These hydrogels remained stable at ambient temperatures and rapidly transitioned to a gel state at body temperature within seconds. Drug release experiments demonstrated the highly controlled release of hydrocortisone from the hydrogel. The temperature increase induced by ultrasound enhances drug concentration as needed. In vivo experiments showed that the selected hydrocortisone mixed micellar hydrogel improved disease outcomes. These findings underscore the potential of ultrasound-responsive hydrogels formed in situ as a promising formulation for the effective treatment of arthritis [32].

Pua et al. designed and developed a poly[4-(2,2,6,6-tetramethylpiperidine-N-oxyl)aminomethylstyrene]-b-poly(ethylene glycol)-b-poly[4-(2,2,6,6-tetramethylpiperidine-N-oxyl)aminomethylstyrene] (PMNT-PEG-PMNT) triblock copolymer for improving the local retention time of ROS scavengers in treating local inflammations like arthritis and periodontal disease. This copolymer utilizes nitrogen oxygen free radical scavenging ROS as side chains and forms a redox-active injectable hydrogel (RIG). The cationic PMNT chain segment in PMNT-PEG-PMNT interacts with anionic polyacrylic acid (PAAc), forming flower-shaped micelles approximately 79 nm in length. In situ thermal irreversible gelation occurs under physiological conditions (Figure 6g). Experimental data indicate that upon the subcutaneous injection of the flower microenvironment solution, RIG not only forms in situ but also demonstrates prolonged retention time at the local injection site. These findings suggest that RIG effectively reduces acute local inflammation associated with oxidative stress and holds promise as an innovative approach for treating local inflammation (Figure 6h,i) [33].

### 2.4. Enzyme-Responsive Hydrogel

Many diseases, including inflammation, infection, and cancer, are often linked to elevated concentrations of various enzymes [59]. A hallmark of OA is the damage and degradation of articular cartilage, primarily due to the excessive production of degrading enzymes. Among these, MMPs are particularly prominent and represent some of the key enzymes in the pathological environment of OA [60]. Based on the overexpression and high activity of MMPs, researchers have developed numerous MMP-responsive drug hydrogel delivery platforms aimed at treating OA [61].

**Figure 7:**
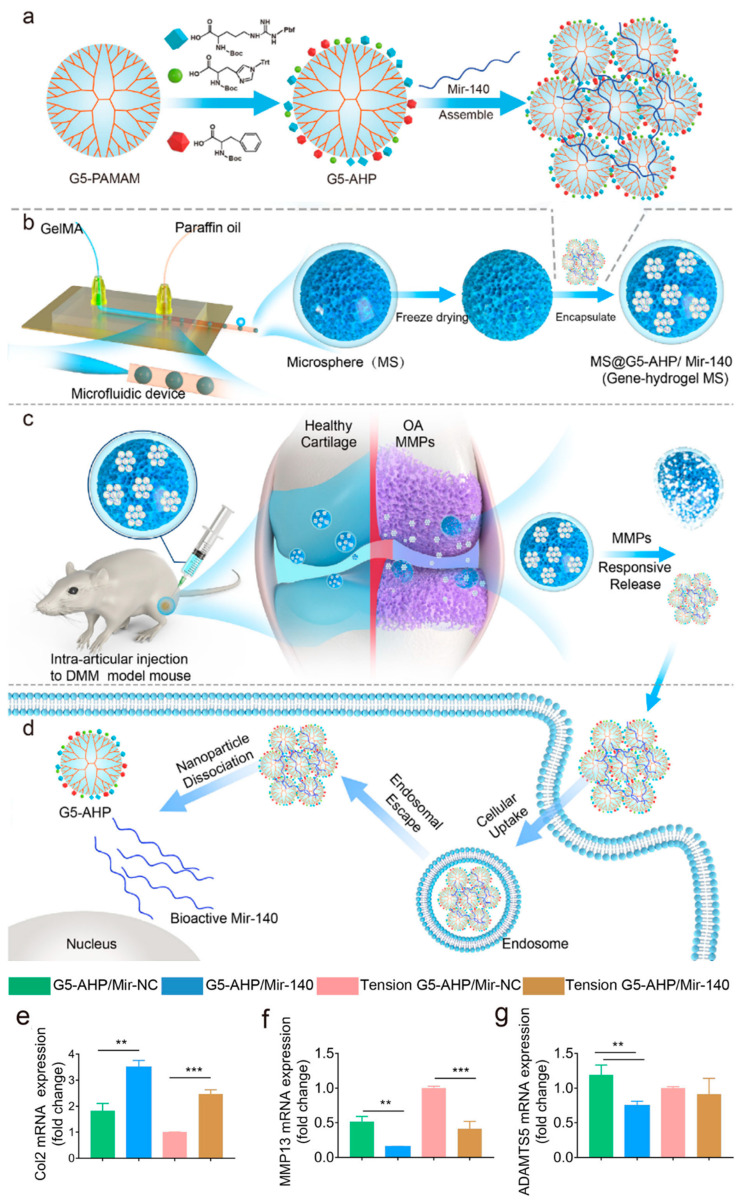
Gene-hydrogel microspheres for the treatment of OA. (**a**) Synthesis of G5-AHP and G5-AHP/miR-140. (**b**) Gene-hydrogel MSs. (**c**) MS@G5-AHP/miR140 was injected into the articular space to alleviate the progression of OA. (**d**) Endocytosis of G5-AHP/miR-140 polyplexes and the release of miR-140. (**e**–**g**) qRT-PCR analyses indicated the mRNA expression of COL2, MMP13 and Adamts5 in chondrocytes (** *p* < 0.01, *** *p* < 0.001.) [34]. Copyright 2022 nature.

Li et al. prepared G5-AHP/miR-140 nanoparticles and loaded them into injectable GelMA-based MMP-responsive HMS (Figure 7a–d). Experimental results showed that after the “nano-micron” composite gene hydrogel was injected into the joint cavity of an OA model, the HMS maintained the long-term release of G5-AHP/miR-140 nanoparticles in response to MMPs. The G5-AHP/miR-140 nanoparticles increased the expression of COL2 (Figure 7e) in OA chondrocytes, reduced the expression of MMPs (Figure 7f) and ADAMTS (Figure 7g), alleviated cartilage matrix degradation, prevented articular cartilage breakdown, and reduced osteophyte formation in surgery-induced OA mice. This study provided a novel cell-free approach to mitigate the progression of OA, demonstrating the potential of local injection gene delivery systems [34].

**Figure 8:**
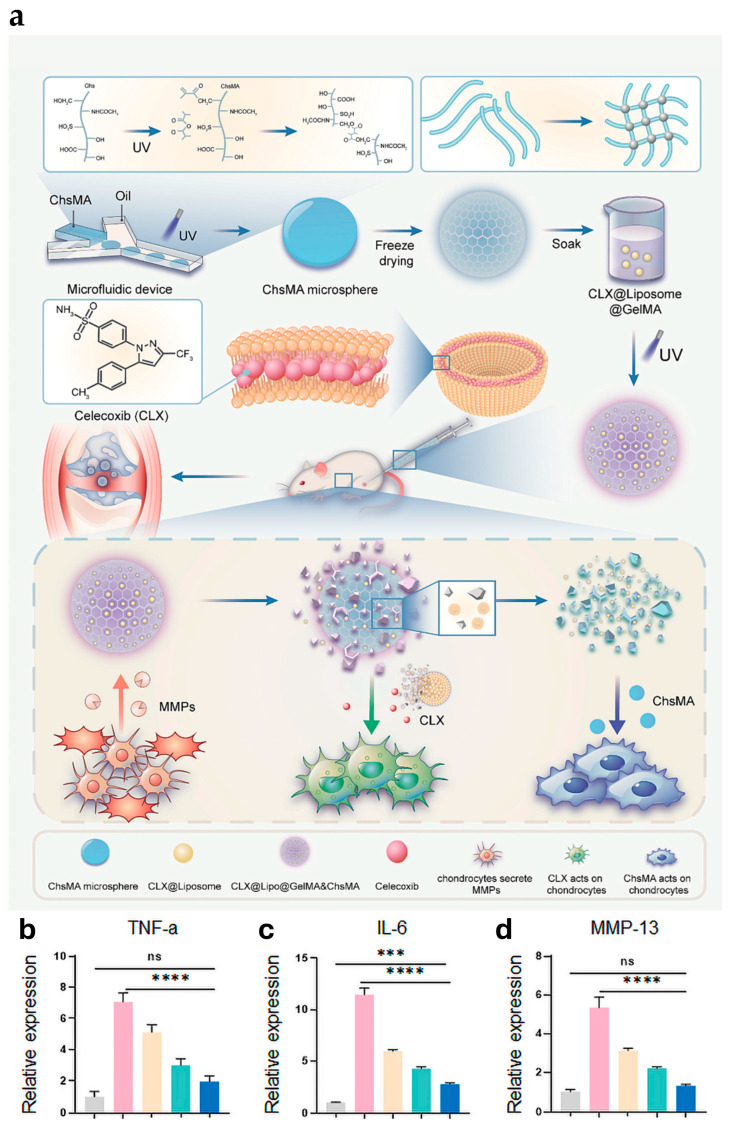
(**a**) Schematic diagram of manufacturing ChsMA+CLX@Lipo@GelMA bilayer microspheres and the effect mechanism after intraarticular injection. The outer layer (GelMA) rapidly degrades in response to MMPs in the OA microenvironment, releasing CLX-loaded liposomes. The released CLX exerts an anti-inflammatory effect, and the inner core structure containing ChsMA microspheres is exposed and begins degrading, promoting the repair of OA cartilage that has undergone degenerative injury. (**b**–**d**) The mRNA expression levels of TNF-α, IL-6 and MMP-13 in IL-1β-treated chondrocytes co-cultured with ChsMA, CLX@Lipo@GelMA, and ChsMA+CLX@Lipo@ GelMA hydrogel microspheres for 24 h analyzed by qRT-PCR (*** *p* < 0.001, **** *p* < 0.0001, ns: represents no significance compared to the PBS and IL-1β groups) [35]. Copyright 2024 Elsevier.

Inspired by the dissolution of the shell structure of chocolate peanuts, Miao designed double-layer microspheres (ChsMA) using microfluidic technology and CLX@Lipo@GelMA, which could respond to the OA microenvironment. First, celecoxib (CLX) was encapsulated into liposomes using the thin film dispersion method. Next, chondroitin sulfate methacryloyl (ChsMA) microspheres were prepared using microfluidic technology. Finally, the microspheres were freeze-dried and soaked in a CLX@Lipo@GelMA solution (Figure 8a). The composition of the outer and inner layers is different: the outer GelMA can react and rapidly degrade in the presence of MMPs in the microenvironment, releasing liposomes loaded with CLX. The released CLX exerts anti-inflammatory effects after the outer layer degrades (Figure 8b–d), while the core structure containing ChsMA microspheres is exposed and begins to degrade, releasing chondroitin sulfate. This promotes the repair of OA cartilage that has undergone degenerative damage. Based on changes in MMPs in the OA microenvironment, two drugs can be intelligently released sequentially to achieve better treatment effects. Therefore, the double-layer HMS were designed to improve responsiveness to the disease microenvironment, making the microspheres more intelligent in disease treatment and showing good application prospects [35].

**Figure 9:**
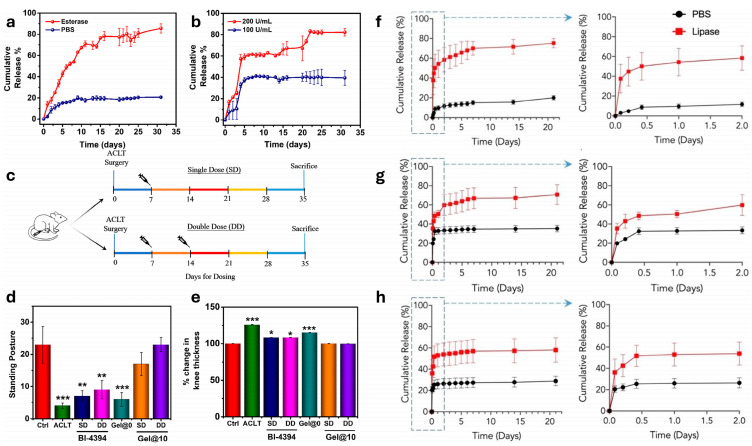
(**a**) BI-4394 release from Gel@10 in the presence and absence of esterase (200 U mL^−1^); (**b**) esterase dependent BI-4394 release from Gel@10. (**c**) Dosing pattern for the treatments; (**d**) quantification of standing position on their hindleg after treatment. (**e**) % Change in the knee thickness of rat after treatment (*** *p* ≤ 0.0001, ** *p* ≤ 0.01 and * *p* ≤ 0.05) [36]. Copyright 2024 Royal Society of Chemistry. Sustained release and enzyme-responsive release of zingerone from TGMS hydrogels encapsulated with (**f**) 5 mg/mL, (**g**) 10 mg/mL, and (**h**) 20 mg/mL of zingerone. For each panel, first 2 days release was plotted separately (right side panels) [37]. Copyright 2022 wiley.

Himadri Shekhar Roy proposed an enzyme-responsive hydrogel capable of continuously releasing 1H-Indole-2-carboxylic acid, 5-[3-[[[(4-carboxyphenyl)methyl]amino]carbonyl]-1-methyl-1H-pyrazol-5-yl]-, 2-ethyl ester (BI-4394) on demand. BI-4394 is reported to block MMP-13 with high specificity and effectiveness [62]. A stable and compatible hydrogel was prepared using glycerol monostearate TG-18. This hydrogel included an enzymatically cleavable linker, which decomposed in the presence of enzymes found in inflammatory environments (Figure 9a), supporting the continuous release of drugs in accordance with the severity of inflammatory conditions (Figure 9b). This approach optimizes treatment effects while reducing toxicity.

The efficacy of the hydrogel in preventing cartilage injury was evaluated in a rat model of anterior cruciate ligament rupture (ACLT)-induced OA (Figure 9c). Experimental results showed that, compared to the direct intra-articular injection of free BI-4394, the use of enzyme-responsive hydrogel for BI-4394 injection was more effective in protecting cartilage from degradation (Figure 9d,e). These findings confirmed the potential role of matrix metalloproteinase-13 inhibitors in preventing cartilage degeneration. Overall, this study demonstrated the promise of using enzyme-responsive hydrogel to deliver BI-4394, presenting a viable treatment option for early-stage OA by preventing further cartilage degradation [36].

Omprakash Sunnapu has developed an encapsulated self-assembled triglyceride monostearate (TGMS) hydrogel loaded with zingerone. This gel used TGMS, and is solid, mechanically stable, and injectable, making it suitable for potential in vivo applications. The conditions were systematically optimized to encapsulate an appropriate dosage of zingerone in the TGMS hydrogel, minimizing non-specific release. Experiments have demonstrated that the TGMS hydrogel encapsulated with zingerone can respond to inflammation-related proteases and release zingerone (Figure 9f,g), which retains its deodorization performance. This self-assembled TGMS hydrogel platform is designed to deliver the anti-inflammatory drug zingerone. Future research will study the efficacy of TGMS hydrogel encapsulated with zingerone in preclinical OA models in vivo [37].

## 3. Conclusions and Future Outlook

The establishment of injectable responsive hydrogel drug delivery platforms aims to enhance drug absorption efficiency and therapeutic efficacy in the treatment of OA. This review primarily examines recent advancements in injectable responsive hydrogel drug delivery systems for OA therapy. A variety of pH/ROS/thermos/enzyme-responsive hydrogels demonstrate significant application potential in OA treatment due to their unique stimulus-responsive mechanisms. However, each type of hydrogel possesses distinct advantages and disadvantages: pH-responsive hydrogels offer sensitivity and design flexibility for precise drug release, yet they often lack sufficient specificity; ROS-responsive hydrogels are highly regarded in inflammation and tumor therapies for their specificity and dynamic drug release capabilities, although their dependence on ROS concentrations limits broader applications; thermo-responsive hydrogels are advantageous for external thermal regulation owing to their controllability and non-invasive stimulation methods, but their thermal effects in deep tissues are constrained; enzyme-responsive hydrogels show considerable promise in disease-specific treatments due to their high specificity and biodegradability, though they are limited by variability in enzyme activity and slower response times. The strengths and weaknesses of different types of responsive hydrogels vary across specific applications, necessitating future research to optimize material design based on particular pathological environments and treatment requirements. By integrating multiple response mechanisms into one hydrogel platform, the therapeutic efficacy and safety of these materials can be enhanced via the synergistic effect. With ongoing improvements in response mechanisms and experimental techniques, targeted and responsive biomaterials hold promise as breakthrough solutions for patients with OA.

## Figures and Tables

**Table 1:** Responsive biomaterials for OA treatment.

Responsive Stimulus	Materials	Responsive Components	Drugs	Effects	Reference
pH	HA/PRP/BM	Schiff base	BM/PRP	Releasing antioxidant BM and cartilage growth promoting PRP on demand	[26]
	IA-ZIF-8@HMs	ZIF-8	IA	Prolonging drug release duration and downregulating the expression of inflammatory factors.	[27]
ROS	mPEG-TK-GLX@PVA-MMA	Disulfide bond	GLX	Inhibiting the expression of NOX4 and reducing the ROS production, thereby mitigating oxidative stress-induced damage.	[28]
	KGN/Dex-TSPBA@WHMs	Phenylboronic acid	KGN/Dex	Dex improves cartilage wear and reduces the expression of TNF-α and IL-6, while WYRGRL has cartilage-targeting capabilities, facilitating better drug delivery to cartilage.	[29]
	Hydrogel@iMVs(Hydrogel: APBA-SF-PVA)	Phenylboronic acid	IFN-γ	Improving mitochondrial dysfunction, reducing the accumulation of reactive oxygen species, and ultimately alleviating cellular senescence.	[30]
Thermo	Rapamycin-loaded P-HA hydrogel	Poloxamer 407	Rapamycin	Inducing the polarization of macrophages from the pro-inflammatory M1 phenotype to the anti-inflammatory M2 phenotype, thereby significantly preventing cartilage degradation.	[31]
	Pluronic F-127/HA/Gelatin	Pluronic F-127	Hydrocortison	Controlling release of the drug by ultrasound-induced vibration and heating.	[32]
	PAAc/PMNT-PEG-PMNT	PMNT/PAAc flower-like micelle	TEMPO	Scavenging ROS to prevent oxidative stress and reducing the expression of TNF-α and IL-1.	[33]
Enzyme	MS@G5-AHP/miR-140	GelMA	miR-140	MiR-140 increased the expression of COL2, reduced the expression of MMPs and ADAMTS, alleviated cartilage matrix degradation, and prevented joint cartilage destruction.	[34]
	ChsMA/CLX@Lipo@GelMA	GelMA	CLX/ChsMA	The double-layer microspheres encapsulate two drugs, with the outer layer releasing medication in response to the inflammatory microenvironment, while the inner layer slowly releases the targeted therapeutic drug.	[35]
	TGMS@BI-4394	TGMS	BI-4394	Blocking the biological activity of MMP-13 protects cartilage from degeneration	[36]
	TGMS/Zingerone	TGMS	Zingerone	Prolonging the retention time of curcumin at the OA site.	[37]

## Data Availability

Not applicable.
